# AGAP2-AS1 May Promote the Occurrence and Development of Glioblastoma by Sponging miR-9-5p: Evidence From a ceRNA Network

**DOI:** 10.3389/fonc.2021.607989

**Published:** 2021-04-06

**Authors:** Xiaobin Luo, Tianqi Tu, Yali Zhong, Shangyi Xu, Xiangzhou Chen, Ligang Chen, Fubing Yang

**Affiliations:** ^1^Department of Neurosurgery, The Affiliated Hospital of Southwest Medical University, Luzhou, China; ^2^Graduate School of Guizhou University of Traditional Chinese Medicine, Guiyang, China; ^3^Sichuan Clinical Research Center for Neurosurgery, The Affiliated Hospital of Southwest Medical University, Luzhou, China; ^4^Academician (Expert) Workstation of Sichuan Province, The Affiliated Hospital of Southwest Medical University, Luzhou, China; ^5^Laboratory of Neurological Diseases and Brain Function, The Affiliated Hospital of Southwest Medical University, Luzhou, China

**Keywords:** bioinformatics analysis, therapeutic targets, glioblastoma, competing endogenous RNA, miRNA, lncRNA

## Abstract

Glioblastoma (GBM), the primary malignant brain tumor, is typically associated with a poor prognosis and poor quality of life, mainly due to the lack of early diagnostic biomarkers and therapeutic targets. However, gene sequencing technologies and bioinformatics analysis are currently being actively utilized to explore potential targets for the diagnosis and management of malignancy. Herein, based on a variety of bioinformatics tools for the reverse prediction of target genes associated with the prognosis of GBM, a ceRNA network of AGAP2-AS1-miR-9-5p-MMP2/MMP9 was constructed, and a potential therapeutic target for GBM was identified. Enrichment analysis predicted that the ceRNA regulatory network participates in the processes of cell proliferation, differentiation, and migration.

## Introduction

Glioblastoma (GBM) is the most common malignant primary brain tumor in the central nervous system (CNS) (1). The associated morbidity results in immense economic burden and medical pressure in the US and around the world (2). Meanwhile, the progressive decline in neurological function and quality of life that results from GBM could have a devastating impact on patients themselves as well as their caregivers and families (3).

For decades, advances have been made in multimodality therapy for GBM, incorporating surgery, radiotherapy, chemotherapy, and targeted therapy as well as systemic therapy and supportive care (1). However, the overall prognosis of patients with GBM remains poor, and the struggle for long-term survival remains a challenge. Notably, an increasing number of studies have reported that non-coding RNA (ncRNA) might play a significant role in pathologic processes, including tumorigenesis and the development of multiple malignant tumors (4). In line with this, there is a hypothesis that the competing endogenous RNA (ceRNA) holds that the long non-coding RNAs (lncRNAs) can sponge and thus inactivate microRNAs (miRNAs) that would otherwise target specific mRNAs for degradation or translational silencing, thus affecting the encoding of proteins (5). By acting as miRNA sponges, lncRNAs regulate the expression levels of the targeted mRNAs, thereby affecting the biological behavior and pathologic process of cancer cells. Therefore, we speculate that the expression of miRNAs must be negatively correlated with that of lncRNAs and mRNAs. Based on the ceRNA hypothesis, the importance of lncRNAs in tumorigenesis becomes more apparent. However, the roles of the ceRNA network of lncRNA–mRNA–miRNA in GBM remain unclear.

Currently, high-throughput (HT) gene sequencing technology has developed rapidly, and online public databases have been widely established. Thus, the resource platforms for data mining and researching provide us with the possibility of a deeper exploration of the molecular mechanisms of diseases. This study used the ceRNA network to strengthen the understanding of the pathogenesis of GBM, and reveal promising therapeutic targets for this malignancy.

## Materials and Methods

### Dataset Collection and Processing

To identify differentially expressed genes (DEGs) between glioblastoma tissues and normal tissues, gene expression profiles GSE90886 (including 9 tumor and 9 non-tumor samples) (6) were downloaded from the Gene Expression Omnibus (GEO, www.ncbi.nlm.nih.gov/geo/) database (7). Moreover, to verify the initial findings, TCGA data from 169 GBM cases and five normal samples were also analyzed. Gene expression profiling was annotated using the annotation documents of the corresponding platforms. If one gene corresponded to multiple probes, the average was used as the gene expression value. Subsequently, the “LIMMA” package in R (version 3.6.2) was used to identify differentially expressed mRNAs between glioblastoma tissues and normal tissues in both GEO datasets and TCGA datasets, and an adjusted *p* < 0.05 and absolute value of log2 fold change |log2 FC| ≥ 2 were considered as the cut-off criteria (8). Then, the differentially expressed mRNAs that were dysregulated in both the GEO and TCGA datasets were divided into upregulated and downregulated groups, and selected for the next analysis. Venn diagrams were plotted using the online tool Venn Diagram (http://bioinformatics.psb.ugent.be/webtools/Venn/).

### Functional Enrichment Analysis and Identification of Hub Genes

To explore the biological functions of the differentially expressed mRNAs, Gene Ontology (GO) annotation and Kyoto Encyclopedia of Genes and Genomes (KEGG) pathway analyses were performed by the “clusterProfiler” package in the R software (9); only the terms with a *p* < 0.05 were considered statistically significant and visualized using the “ggplot2” package in R (10). To identify the key genes correlated with the prognosis of patients with GBM, the prognostic values of the dysregulated genes were evaluated using clinical information obtained from the TCGA database. Subsequently, these values were evaluated using the Cox proportional-hazards model. For the upregulated group, only genes with a hazard ratio (HR) >1 and a *p* < 0.05 were considered statistically significant, while for the downregulated group, the threshold was set at HR <1 and a *p* < 0.05.

### Prediction of Upstream miRNAs

The upstream miRNAs that regulate the expression of key genes were predicted using miRTarbase (http://mirtarbase.cuhk.edu.cn/php/index.php), a web-based database that has accumulated more than 360,000 miRNA-target interactions (MTIs) validated experimentally by reporter assay, western blot, microarray, and next-generation sequencing experiments (11). To enhance the reliability of the predicted results, only the interactions with strong evidence (reporter assay, western blot, and qPCR) were included. Subsequently, a survival analysis of miRNA was performed using the oncoLnc database, GBM samples from TCGA were ranked for miR-9-5p expression; 140 samples in the top 25% expression range and 140 samples in the bottom 25% expression range were compared for possible differences in survival, then we downloaded the expression of glioblastoma miRNA. Moreover, to assess miRNA expression in non-GBM samples, the collated miRNA matrix data were downloaded from the Firehose database (https://gdac.broadinstitute.org/). The data of the non-GBM sample miRNA expression were extracted, and GraphPad Prism (version 8.3.0, https://www.graphpad.com/) was used for difference analysis. A *p* < 0.05 was considered statistically significant.

### Prediction of Upstream lncRNA

The upstream potential lncRNAs interacting with key miRNAs were predicted using the TargetScan (http://www.targetscan.org/) (12) and LncBase v2 (www.microrna.gr/LncBase) (13) databases. To enhance the reliability of the predicted results, only the upstream lncRNAs predicted by both databases were included. Subsequently, the expression levels and prognostic values of the predicted lncRNA for overall survival in GBM patients were evaluated using Gene Expression Profiling Interactive Analysis (GEPIA; http://gepia.cancer-pku.cn/detail.php), a newly developed interactive web server for analyzing the RNA sequencing expression data of 9,736 tumors and 8,587 normal samples from the TCGA and GTEx projects (14). A *p* < 0.05 was considered statistically significant.

### Construction of the lncRNA-miRNA-mRNA Regulatory Network and Functional Annotation

Based on the prediction and evaluation above, a novel lncRNA-miRNA-mRNA regulatory network associated with the prognosis of patients with GBM was constructed and visualized using the Cytoscape software (v3.7.2, http://www.cytoscape.org/) (15). In addition, the Database for Annotation, Visualization, and Integrated Discovery (DAVID v6.8 https://david.ncifcrf.gov/home.jsp) (16) bioinformatics database was used to explore the biological function of this ceRNA regulatory network in human GBM. Only the terms with a *p* < 0.05 were considered statistically significant.

## Results

### Identification of Differentially Expressed mRNAs in GBM

**Figure 1:**
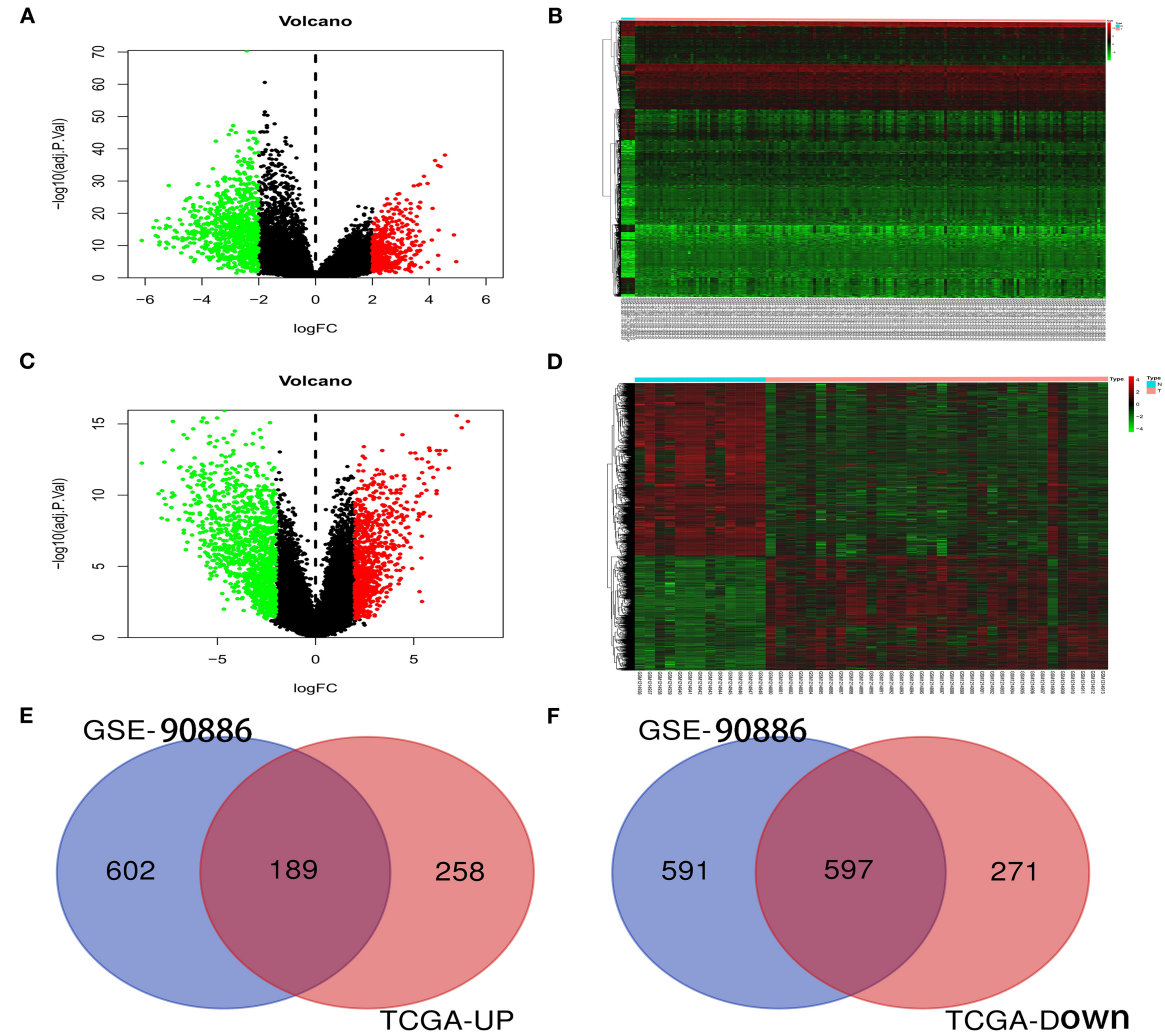
Identification of differentially expressed genes. **(A,C)** The volcano plots of DEGs in TCGA and GSE90886 datasets. Green and red represent the significantly downregulated and upregulated genes, respectively. The black dot represents genes with no significant difference. **(B,D)** The heat maps of the expression of DEGs in the TCGA and GSE90886 datasets. **(E,F)** The intersection of upregulated and downregulated genes in the 2 datasets, respectively. DEG, Differentially expressed gene.

In the GSE90886 dataset, 1979 differentially expressed mRNAs (including 791 upregulated and 1,188 downregulated genes) were screened out (Figures 1A,B). Meanwhile, 1,315 differentially expressed mRNAs (including 447 upregulated and 868 downregulated genes) were also identified in the TCGA dataset (Figures 1C,D). After the upregulated or downregulated mRNAs in the 2 datasets were intersected separately, 189 upregulated and 597 downregulated differentially expressed genes were identified that appeared in both datasets (Figures 1E,F, Supplementary Tables 1, 2).

### Functional Enrichment Analysis of Differentially Expressed mRNAs

**Figure 2:**
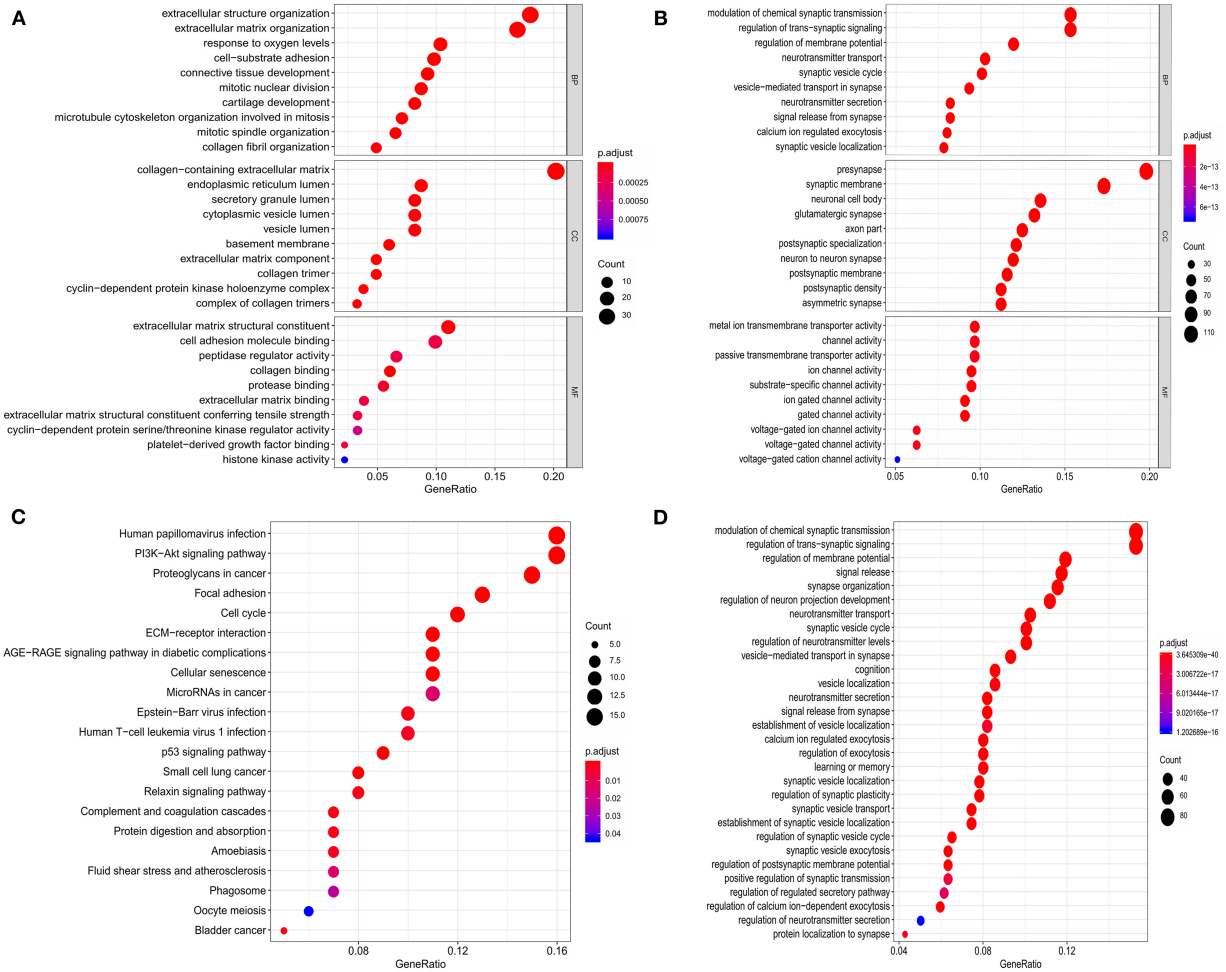
Functional enrichment analysis of the DEGs. **(A,B)** GO enrichment analysis of the upregulated and downregulated DEGs, respectively. **(C,D)** KEGG pathway enrichment analysis of the upregulated and downregulated DEGs, respectively.

Functional and pathway enrichment analyses of the identified genes were performed using the “clusterProfiler” package in R. As shown in Figures 2A,B, the GO analysis results showed that for biological processes (BP), upregulated DEGs were mainly enriched in extracellular structure organization, response to oxygen levels, and cell-substrate adhesion; downregulated DEGs were mainly enriched in modulation of chemical synaptic transmission, regulation of trans-synaptic signaling, and regulation of membrane potential. For cell components (CC), upregulated DEGs were mainly enriched in the collagen-containing extracellular matrix, endoplasmic reticulum lumen, and secretory granule lumen; downregulated DEGs were mainly enriched in pre-synapse, synaptic membrane, and neuronal cell body. For molecular function (MF), upregulated DEGs were mainly enriched in extracellular matrix structural constituents, cell adhesion molecule binding, and peptidase regulator activity; downregulated DEGs were mainly enriched in metal ion transmembrane transporter activity, channel activity, and passive transmembrane transporter activity. The enriched KEGG pathways of the DEGs are shown in Figures 2C,D. The upregulated DEGs were mainly enriched in the P53 signaling pathway, PI3K–Akt signaling pathway, proteoglycans in cancer, focal adhesion, and cell cycle; downregulated DEGs were mainly enriched in the modulation of chemical synaptic transmission, regulation of trans-synaptic signaling, and regulation of membrane potential.

### Identification of Hub Genes and Upstream miRNAs

**Figure 3:**
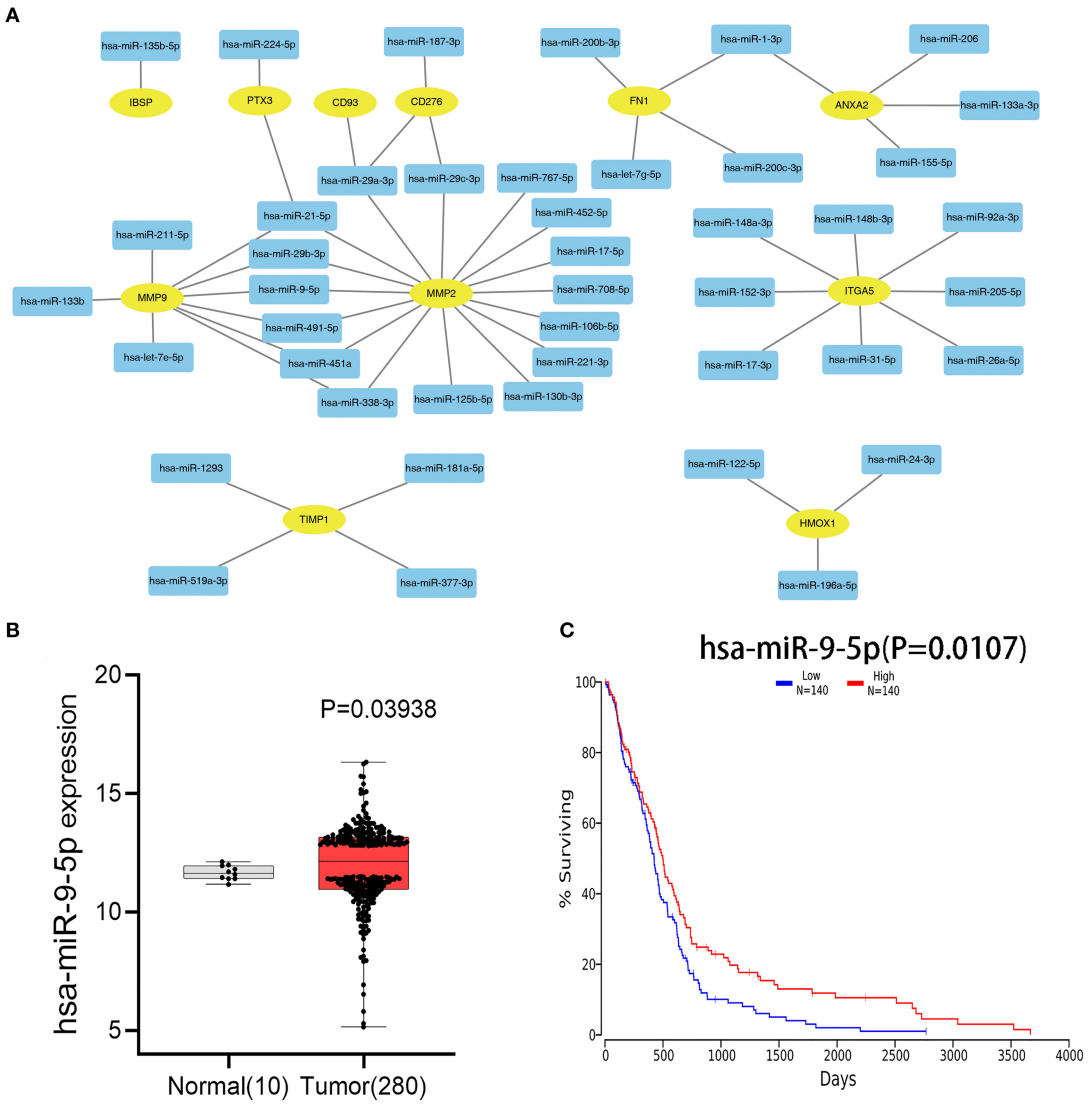
Identification of upstream miRNAs. **(A)** The mRNA–miRNA network constructed by cytoscape software. The rectangle in the network represents miRNA, and the ellipse represents hub genes. **(B)** Differential analysis of miR-9-5p gene expression between Tumor (GBM) group and normal brain tissue group. **(C)** The prognostic values of miR-9-5p in patients with GBM.

After combining the expression levels and prognostic values, 24 upregulated DEGs were found to not only be dramatically upregulated in GBM but also significantly associated with poor prognosis of patients with GBM. Only one downregulated DEG showed low expression and was associated with improved prognosis in patients with GBM (Table 1). Subsequently, these 25 survival-related DEGs were considered as key genes in GBM and selected for further study. To identify key miRNAs that regulate these hub genes, the upstream miRNAs of the 25 survival-related DEGs were predicted using the experimentally validated miRNA-target interactions database, miRTarBase. As a result, 44 miRNAs were identified to regulate 11 survival-related DEGs (IBSP, PTX3, CD93, CD276, FN1, ANXA2, MMP9, MMP2, ITGA5, TIMP1, and HMOX1) (Figure 3A). The other 14 survival-related DEGs were not targeted by any miRNA in this database. The expression levels and prognostic values of the predicted miRNAs were further evaluated for overall survival in GBM patients using the Firehose and OncoLnc databases, respectively. Figures 3B,C indicates that the comparison of miRNA expression levels and clinical data identified a correlation between the low expression of miR-9-5p and poor prognosis in GBM. This miRNA was identified as a key upstream miRNA and selected for subsequent analysis.

**Table 1:** Survival analysis of the DEGs.

	**Gene symbol**	**HR**	**HR95L**	**HR95H**	***P*-value**
Up-regulated	MMP2	1.010541032	1.004949241	1.016163936	0.000212369
	ANXA2	1.005889372	1.000450616	1.011357695	0.033768093
	ITGA5	1.024931506	1.007056596	1.043123689	0.006082391
	C1R	1.00428692	1.000789627	1.007796434	0.016241772
	HMOX1	1.001555783	1.00001654	1.003097395	0.047586642
	CD276	1.017291089	1.00291214	1.031876192	0.01825859
	POSTN	1.002754778	1.00052993	1.004984574	0.015205621
	CFI	1.016857435	1.001684154	1.032260557	0.029306594
	STEAP3	1.013789666	1.003281262	1.024408135	0.009990071
	MDK	1.005749243	1.001945295	1.009567633	0.003025407
	CHI3L1	1.000138279	1.000030203	1.000246366	0.012150438
	GAL3ST4	1.02174166	1.003854776	1.039947256	0.016989945
	SERPINA1	1.014860392	1.00525365	1.024558941	0.002367752
	FN1	1.001644372	1.000138786	1.003152224	0.032291479
	CD248	1.015844372	1.004990377	1.026815591	0.004127917
	CD93	1.024640297	1.000658572	1.049196768	0.043962554
	PTX3	1.00847434	1.003710168	1.013261124	0.00047804
	PDIA4	1.006520286	1.001741253	1.01132212	0.007441631
	TIMP1	1.000848975	1.000441049	1.001257068	0.000045
	TNFRSF12A	1.006104895	1.000027971	1.012218747	0.048951623
	IBSP	1.009347246	1.000701291	1.0180679	0.034032832
	S100A11	1.001305193	1.00009462	1.002517232	0.034578804
	SPOCD1	1.013555775	1.001458828	1.025798844	0.027954563
	MMP9	1.005911774	1.000336973	1.011517642	0.037637096
Down-regulated	DPP10	0.839069186	0.72989918	0.964567598	0.013616218

### Identification of Upstream lncRNAs

**Figure 4:**
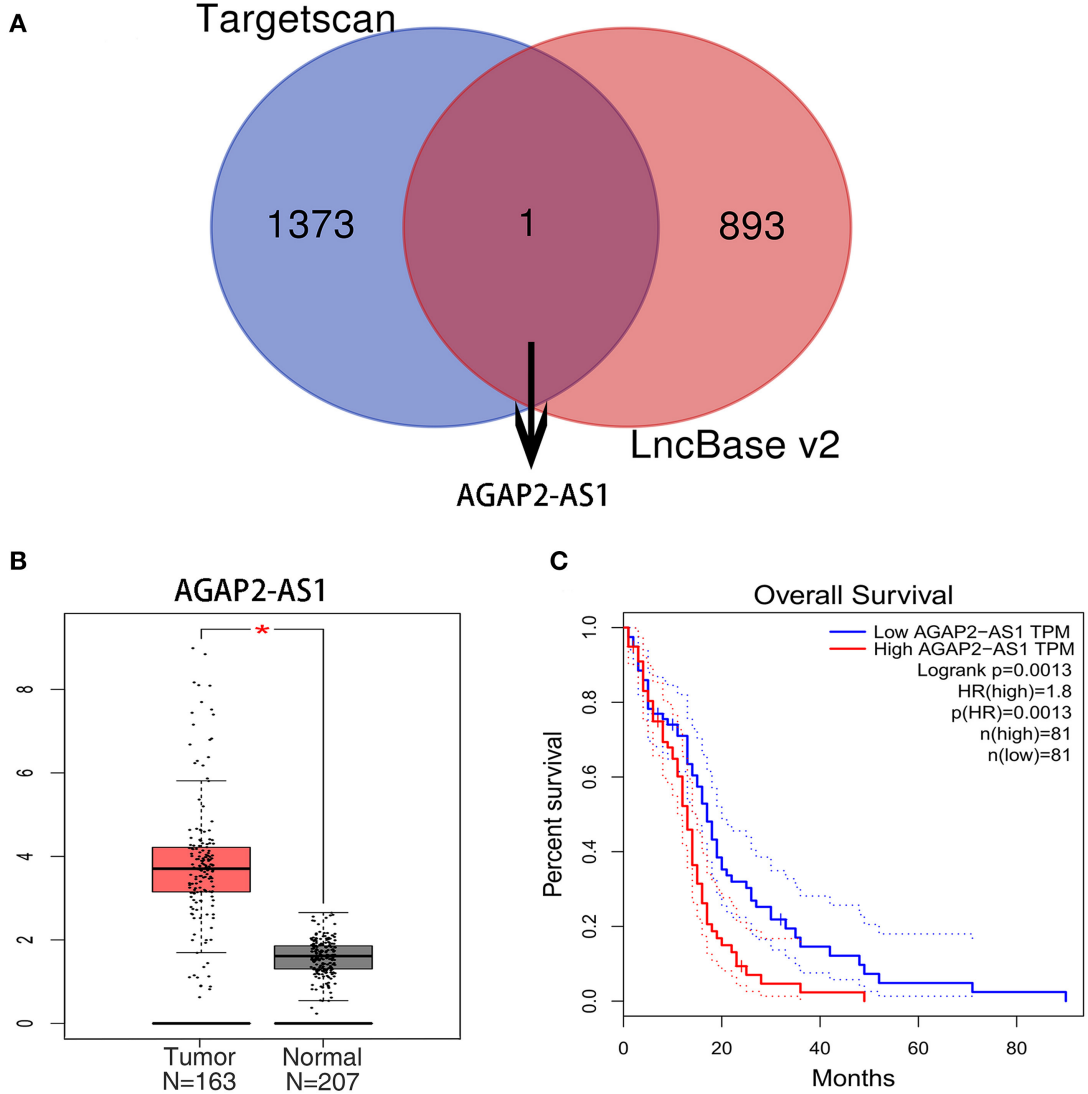
Identification of upstream lncRNAs. **(A)** The lncRNA targeted by miR-9-5p in the Targetscan and LncBase v2 databases. **(B)** The expression level of AGAP2-AS1 between GBM and normal samples in the GEPIA database. The asterisk indicates that the difference between the Tumor (GBM) group and the Normal brain tissue group is statistically significant (*P* < 0.05). **(C)** The prognostic value of AGAP2-AS1 in human GBM. TPM is the abbreviation of Transcripts Per Million, which is the standardized statistic used in RNA-SEQ data analysis, and its dotted lines represent the 95% confidence interval.

The upstream potential lncRNAs that interact with key miRNAs were predicted using the TargetScan and LncBase v2 databases. As a result, one upstream lncRNA was predicted to regulate miR-9-5p (Figure 4A). Based on the ceRNA hypothesis, the eligible lncRNAs should be negatively correlated with miRNA and positively correlated with mRNA. Therefore, the expression levels and prognostic values of these upstream lncRNAs were assessed using the GEPIA database. As a result, a comparison of lncRNA expression and clinical data identified a correlation between high levels of AGAP2-AS1 and worse prognosis (Figures 4B,C). AGAP2-AS1 was considered a key upstream lncRNA and was chosen for subsequent analysis.

### Integration of the Survival-Related ceRNA Regulatory Network and Functional Annotation

**Figure 5:**
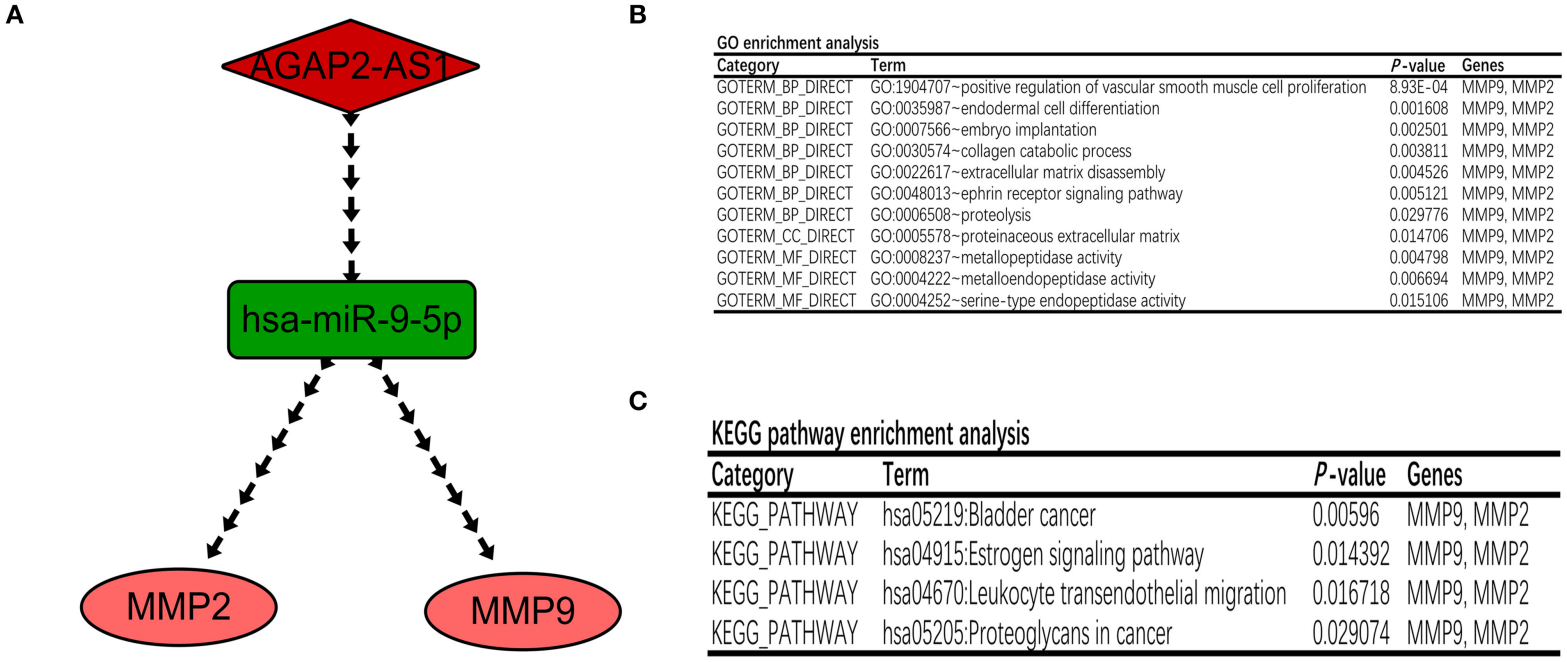
Construction of a GBM-related lncRNA–miRNA–mRNA regulatory network. **(A)** The ceRNA network associated with GBM prognosis. The red diamond represents the key lncRNA, the green rectangle represents the key miRNA, and the pink ellipse represents the key mRNA. **(B,C)** GO and KEGG enrichment analyses of genes in the ceRNA regulatory network.

Based on the prediction and validation above, a survival-related lncRNA-miRNA-mRNA ceRNA regulatory network consisting of one lncRNA-miRNA pair (AGAP2-AS1-miR-9-5p) and two miRNA-mRNA pairs (miR-9-5p-MMP2/MMP9) was constructed and visualized using Cytoscape (Figure 5A). Every gene in the ceRNA regulatory group was associated with the prognosis of patients with GBM. Importantly, each regulatory relationship in the present ceRNA network was fully consistent with the ceRNA mechanism. In addition, the DAVID database was used to explore the biological functions of the ceRNA regulatory network. A GO enrichment analysis predicted that the ceRNA regulatory network participates in the processes of cell proliferation, cell differentiation, and cell migration (Figure 5B). KEGG pathway enrichment analysis revealed that the ceRNA regulatory network was mainly enriched in bladder cancer, the estrogen signaling pathway, leukocyte transendothelial migration, and proteoglycans in cancer (Figure 5C).

## Discussion

GBM is a rare disease, with non-specific symptoms, therefore, it is difficult to diagnose at its early stages (17). In recent years, with the rapid development of examination and treatment technologies, the detection rate of GBM has greatly improved, and patient survival has been prolonged to some extent. However, the potential molecular mechanism underlying the initiation and promotion stages of GBM remains to be elucidated, and early diagnostic biomarkers and therapeutic targets still need to be explored.

Accumulated evidence has shown that lncRNAs act as miRNA sponges and participate in multiple pathological processes, including tumor formation, development, and evolution. For example, in a renal cell carcinoma model, Zhai et al. indicated that lncRNA-SARCC could suppress renal cell carcinoma (RCC) progression by altering androgen receptor (AR)/miRNA-143-3p signals (18). Li et al. concluded through analysis that H19, a long non-coding RNA, promotes gastric carcinogenesis by sponging miR-29a-3p (19). Yu et al. reported that lncRNA-CCAT2 regulates miR-145 expression by suppressing its maturation process in colon cancer cells (20). In addition, a growing body of evidence has also indicated that lncRNA-related ceRNA networks could play a crucial role in the initiation and promotion of GBM. For instance, Wang et al. demonstrated that the inhibition of COX-2, mPGES-1, and CYP4A blocks angiogenic Akt signaling in gliomas through the ceRNA effect of miR-194-5p and lncRNA NEAT1 (21). Another study reported that, through the regulation of ADAM12, SP1-mediated upregulation of lncRNA LINC01614 functions as a ceRNA for miR-383, thereby facilitating glioma progression (22). Using a comprehensive transcriptomic analysis and experimental validation, Chen et al. identified lncRNA HOXA-AS2/miR-184/COL6A2 as the critical ceRNA regulatory complex involved in low-grade glioma recurrence (23).

Then, through prediction and stepwise reverse validation from mRNA to lncRNA, which were validated using multiple databases, the upstream miRNAs and lncRNAs were identified. Moreover, a novel ceRNA network, consisting of GBM-related lncRNA (AGAP2-AS1), miRNA (hsa-miR-9-5p), and mRNAs (MMP2 and MMP9), was constructed successfully. In addition, all of the key components in this network were not only significantly differentially expressed in GBM vs. normal samples, but also had a marked correlation with prognosis. Meanwhile, all components of this network were fully compliant with the ceRNA hypothesis.

According to the pathway enrichment analysis, the genes in the network were primarily enriched in tumor-related pathways, which means that the network affects many biological behaviors and pathological processes of gliomas. Numerous studies have reported that these pathways are closely associated with the tumorigenesis, development, and evolution of GBM. For example, the PI3K/Akt signaling pathway is reported to be closely related to the proliferation and migration of glioma cells as well as to glioma tumorigenicity (24, 25). Similarly, for the progression of malignant gliomas, the p53-related pathway could be a promising research target, and could also affect glioma stem/progenitor cell renewal and differentiation (26). Meanwhile, evidence has also confirmed that the expression of p53 is related to the prognosis of patients with malignant gliomas (27). Furthermore, the cell cycle is critical for cell growth and cell division, which determine the rate of tumor progression (28–30). The present study also suggested that the ceRNA regulation network has an impact on the cell cycle and consequently influences tumor outcomes.

lncRNAs are classic noncoding RNAs that have been reported to be key regulators of cancer progression. Previous studies have demonstrated the roles of lncRNAs in glioma development (31–33). According to the present study, the expression of AGAP2-AS1 may be related to the prognosis of GBM patients. Similar to these findings, researchers found that the AGAP2-AS1 gene has potent roles in numerous pathologic processes of cancer, including proliferation, invasion, and metastasis. In gastric cancer, AGAP2-AS1 is activated by SP1, promoting cell proliferation and invasion (34). In hepatocellular carcinoma, AGAP2-AS1 is reported to function as a competitive endogenous RNA; it upregulates ANXA11 expression by sponging miR-16-5p and promotes cell proliferation and metastasis (35). In pancreatic cancer, upregulation of the AGAP2-AS1 gene could regulate cell proliferation and migration, partly through the suppression of ANKRD1 and ANGPTL4 (36). In addition, in the research field of GBM, the lncRNA AGAP2-AS1 has also been reported to regulate the tumorigenesis and development of glioma cells. For example, by sponging miR-15a/b-5p to upregulate the expression of HDGF and activating the Wnt/β-catenin signaling pathway, AGAP2-AS1 could promote the proliferation of glioma cells (37).

Evidence has proven that miR-9-5p could be a protagonist in the regulation network of the pathological process of tumors. This key molecule plays a significant role in inhibiting the proliferation and differentiation of tumor cells, thereby interfering with the progression of cancer (38–40). In the GBM cell model, miR-9-5p was shown to inhibit glioma cell proliferation by downregulating FOXP2 (41). Interestingly, in the present study, miR-9-5p was found to be a key regulator in the regulation network, as it could be target-regulated by AGAP2-AS1.

The mRNAs coding for the matrix metallopeptidases MMP2 and MMP9 were identified as potential targets for miR-9-5p in our model. As reported in the literature, MMP2 and MMP9 are closely related to vasculogenic mimicry formation in gliomas (42). Thus, by regulating the MMP2-related pathway, glioma angiogenesis could be inhibited (43). Meanwhile, the regulation of MMP9 activity was shown to be correlated with tumor cell invasion (44). More importantly, glioma cell invasion, migration, and secretion were reported to be a result of the increased expression of MMP2 and MMP9 (45). Thus, it has been gradually accepted that MMP2 and MMP9 are candidate biomarkers for monitoring chemotherapy in high-grade glioma (46) as well as indicating poor prognosis in glioma recurrence (47).

Inevitably, some limitations were present in this study. Since multiple GEO and TCGA datasets were included, the tumor samples were not evaluated for GBM phenotypes, which may have affected the expression profiles and prognosis of GBM patients. Additionally, all survival analyses were based only on GBM samples and performed using the online database, and the confounders were evaluated automatically. It is entirely possible that bias could have occurred. Moreover, the main screening index of this study was the prognostic value, which may have led to the omission of potentially valuable information. Most importantly, all data and results of this study were based on public databases and online bioinformatics tools, so further basic experiments and clinical studies need to be conducted to validate the findings.

## Conclusion

ceRNA regulatory networks involved in the progression of GBM have been reported, while only a few studies have focused on constructing a ceRNA network associated with the prognosis of GBM patients. More importantly, previous studies have constructed GBM-related ceRNA networks mainly via lncRNA–miRNA–mRNA sequential patterns. In contrast, the present study was the first to construct a GBM-related ceRNA network via mRNA–miRNA–lncRNA sequential patterns. In summary, this study revealed a potential novel ceRNA regulatory network whose experimental validation may provide new insights into the pathogenesis of GBM.

## Data Availability Statement

The raw data supporting the conclusions of this article will be made available by the authors, without undue reservation.

## Author Contributions

XL, TT, YZ, SX, XC, LC, and FY designed the study. XL, TT, YZ, SX, XC, and LC performed the bioinformatics analysis and interpretation of the data. XL, TT, and YZ wrote the manuscript. LC and FY revised the manuscript and gave final approval of the version to be published. All authors read and approved the final manuscript.

## Conflict of Interest

The authors declare that the research was conducted in the absence of any commercial or financial relationships that could be construed as a potential conflict of interest.
